# Relationship Quality and Health Among Black Same-Sex Male Couples: Protocol for a Symbolic Netnography Study

**DOI:** 10.2196/29589

**Published:** 2022-06-03

**Authors:** Jonathan Mathias Lassiter, Jagadīśa-devaśrī Dacus, Mallory O Johnson

**Affiliations:** 1 Department of Psychology Rowan University Glassboro, NJ United States; 2 Institute for Sexual and Gender Minority Health and Wellbeing Northwestern University Chicago, IL United States; 3 Department of Medicine University of California, San Francisco San Francisco, CA United States

**Keywords:** symbolic netnography, qualitative, relationship health, Afrocentric psychology, same-sex couples, Black sexual minority men, interdependence theory, methods

## Abstract

**Background:**

Across a range of studies, health scientists have found that being in a romantic relationship can have positive and negative influences on one’s health. A couple’s health outcomes are often influenced by relationship quality—or how they perceive the positive or negative character of their relationship. These findings have important implications for how scientists and interventionists may leverage romantic relationships facilitating good health among couples. However, in general, couples research has not included Black same-sex male couples in large enough numbers to make previous studies’ findings relevant to them. This represents a gap in the scientific literature and, more importantly, a missed opportunity to understand how romantic relationships influence health for a group that must navigate distinct, multilevel health and social inequities.

**Objective:**

This study aims to (1) decode and understand the ways in which Black same-sex male couples express their romantic relationships in virtual contexts via symbolic indicators, (2) determine how Black same-sex male couples describe the quality of their romantic relationships, and (3) explore how Black same-sex male couples make meaning of their relationship quality and its impact on their relational and individual health.

**Methods:**

We will use joint dyadic interviews embedded within a symbolic netnography research design to accomplish our aims. We will use grounded theory to analyze our qualitative data. We will then triangulate our findings to determine how well they answer our research questions.

**Results:**

This study received ethical approval on October 8, 2020 and we began data collection in November 2020. Results are expected to be available no later than December 31, 2022.

**Conclusions:**

This study will apply novel symbolic netnographic qualitative methods to further our understanding of Black same-sex male couples’ romantic relationships and how they contribute to their health. The findings will be used to develop programs to improve Black same-sex male couples’ health in community and virtual settings.

**International Registered Report Identifier (IRRID):**

DERR1-10.2196/29589

## Introduction

### Background

Several psychological and public health studies have found that being in a romantic relationship can have positive and negative influences on the individual health of each partner and the couple as a unit [1-4]. In general, romantic partners mutually influence each other’s health [5], and each romantic partner’s health tends to become more similar over time [6]. These health outcomes are influenced by many factors, several of which are related to relationship quality. Relationship quality has been defined as one’s subjective judgment of the positive or negative character of one’s relationship [7]. Relationship quality encompasses a range of positive feelings and emotions, such as feelings of love, trust, care, affection, and commitment [8]. Social support for one’s relationship has also been found to be a positive influence on relationship quality [9,10].

However, in general, couples research has not included Black same-sex male couples (BSMCs) in large enough numbers to make the available findings relevant to them [11]. This represents a gap in the scientific literature and, more importantly, a missed opportunity to understand how romantic relationships influence health for a group that must navigate distinct, multilevel health and social inequities. The few studies specifically about BSMCs have seldom focused on relationship quality and the factors that influence it outside of the context of HIV prevention [12-14]. For example, we know little about these couples’ relationship intimacy, physical affection, and couple-level sexual orientation disclosure [9,15]. However, these factors may be assets that could be leveraged to promote relationship quality, mitigate health inequities, and promote holistic (ie, biopsychosocial-spiritual) health among BSMCs. Overall, there are several relational determinants that could affect these couples’ relationship quality and health that are understudied or have not yet been identified.

Researchers who investigate these understudied and yet-to-be-identified factors affecting BSMCs’ relationship quality and health will benefit from utilizing culturally specific frameworks that are relevant to future couples-based health interventions and policies for this group [16]. Qualitative methods are an appropriate starting point in developing and refining these culturally specific frameworks [17]. Qualitative methods will allow researchers to understand BSMCs in their own words. Researchers may also learn how these couples characterize and articulate their relationship quality and make meaning of its relationship to their health. We elected to use qualitative methods for these reasons. Specifically, we will use symbolic netnography and joint dyadic interviewing to gain an in-depth understanding of the couples’ relationship quality and health, as well as to refine our culturally specific theoretical framework, described below, to explain how these factors influence each other.

Symbolic netnography is a qualitative research methodology that seeks to understand a group’s cultural experiences as presented via social media [18]. Unlike media analysis, which examines portions of media content, symbolic netnography examines greater portions of communication, information, and culturally representative content that are mediated by technological platforms and applications. Symbolic netnography’s cultural centering involves elucidating cultural elements found in social media communication, such as language, identities, values, and visual imagery (ie, photos, videos, and emoticons). Further, researchers are participant-observers, because they need to have an embedded cultural understanding of the phenomenon; otherwise, their interpretations become more descriptive than explanatory.

### Theoretical Framework

The philosophical assumptions that underpin the present study are informed by an integrated Afrocentric interdependence theoretical framework that merges Optimal Conceptual Theory applied to sexual and gender minorities (OCT-SGM) [19,20], interdependence theory [21], and Wilson and colleagues’ model [22] for Black men’s positive mental health. Specifically, OCT-SGM is an Afrocentric theory that assumes the following [23]:

(a) Life happens in a spiritual context and...human beings are the physical manifestation of spirit (i.e., extrasensory energy that connects all life-forms); (b) good health is achieved through perceptual and behavioral alignment with the spiritual nature of life, (c) the relationships between the interlocking systems of oppression at the societal level interact with sexual and gender minorities (SGMs) intersecting identities at the individual level to confer privilege (e.g., socioeconomic resources) and disadvantage (e.g., intersectional stressors) that vary across time and context, and (d) SGM people’s spiritual alignment and responses to varying levels of privilege and disadvantage mutually influence each other.

From this perspective, spiritual alignment is fluid and can fluctuate from high to low. OCT-SGM is a holistic theory that can help us begin to understand how spirituality influences BSMCs. It should be noted that the spirituality discussed here is culturally specific to Black sexual-minority men and has been defined as follows [23]:

(a) [A] relationship with something greater than themselves, (b) part of themselves, (c) a guiding force in their lives, and (d) multidimensional in nature. The culturally-specific aspects of their spirituality were identified as being (a) an energetic union of masculine and feminine energy within their physical body, (b) connected to their ancestors, (c) an integration of the divine and the sensual, and (d) the use of spirituality to combat intersectional oppression.

This definition of spirituality was developed from qualitative work conducted exclusively with Black sexual-minority men to understand their particular ways of making meaning of the sacred. It is important to note that scholars have conceptualized spirituality as a distinct construct from religion. Religion has been defined as “a shared system of beliefs, mythology, and rituals with a god or gods” [24]. There has been very little research related to the role of spirituality in same-sex couples’ lives. Most of that research has been conducted with predominately white samples [25]. The limited research on this topic has found that religious same-sex couples believe that their religious and spiritual values add sacred meaning to their relationships [26]. Additionally, some couples have reported that they are subjected to the homonegative attitudes of others who use religious doctrine to justify their prejudice. These findings provide couple-level data but have very little relevance for BSMCs. The research studies they are based upon are not inclusive of these couples or grounded in their cultural realities. Our culturally specific framework of OCT-SGM may be applied at the couple level by examining it alongside interdependence theory.

Interdependence theory purports that partners in a couple mutually influence each other and make subjective judgments about those influences related to whether they confer rewards or costs to each individual partner and the couple as a whole [21]. The aim of interdependence theory is to “explain how two partners influence each other, how this shapes couples’ interactions, and how these interactions determine the development of relationships” [21]. Interdependence theory helps make sense of how couples’ individual and dyadic experience of their relationships helps to maintain them or lead to their termination.

Wilson and Williams [27] and Wilson and colleagues [22] proposed that positive mental health outcomes for Black men were tied to their ability to engage and enact consciousness, connection, and competency in their interpersonal relationships. Consciousness refers to one’s level of spiritual consciousness (ie, spiritual alignment). Connection refers to one’s relationships with one’s spiritual nature, the spiritual nature of others, and the expression of the sacred in one’s actions with others. Competency is one’s ability to develop, choose, and effectively implement social, cognitive, behavioral, and affective skills to create and maintain equitable relationships and health. Taken together, positive mental health for Black men is directly related to the quality of their relationships.

**Figure 1:**
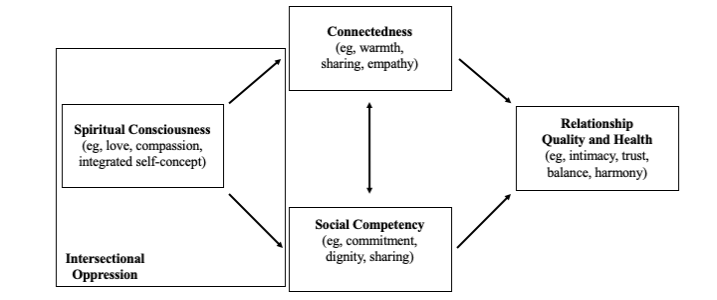
Integrated Afrocentric interdependence theoretical framework.

Our integrated Afrocentric interdependence theoretical framework (Figure 1) proposes that BSMCs’ health, as individual partners and as a unit, is determined by their ability to maintain perceptual consciousness and behavioral alignment with the spiritual nature of life as they navigate varying levels of privilege and disadvantage due to interlocking systems of oppression. These experiences of intersectional oppression have the potential to dampen spiritual consciousness and negatively affect the romantic relationship. These experiences may also help BSMCs rely more on their spiritual consciousness to make meaning of their experiences, both positive and negative, within and outside of the couple, as opportunities to enhance healthy relationship factors. High spiritual consciousness allows BSMCs to cultivate and share cultural strengths (eg, racial pride and adaptability) and develop integrated holistic self-concepts as Black sexual-minority men that contribute to self-love and compassion. This spiritual consciousness affects their ability to achieve substantive connection to themselves and their romantic partners and cultivate warmth, sharing, and empathy in their relationships. Spiritual consciousness also helps these couples cultivate perceptual and social environments that are conducive to developing social, cognitive, behavioral, and affective skills to deepen commitment, dignity, and sharing within their relationships. These factors (ie, spiritual consciousness, connectedness, and social competency) are interrelated, and when well-developed contribute to high relationship quality and good health [27].

We acknowledge that our integrated Afrocentric interdependence theoretical framework is highly conceptual at this point. This is purposeful, as we have attempted to explain the relationships between culturally specific psychosocial-spiritual elements of BSMCs’ health. This level of innovation is necessary for two specific reasons. First, there is simply not enough currently available empirical work on these topics with this particular population. Second, it is imperative that we ground our understanding of BSMCs outside of Eurocentric epistemologies. We intentionally center the Afrocentric paradigm to counter Eurocentric ways of knowing and epistemologies of ignorance that highlight deficit and pathology-related foci and thereby sustain health inequities in their (explicit and implicit) perpetuation of oppressive ideologies [12,28]. Our theoretical framework provides a starting place to understand BSMCs’ health in a way that is culturally grounded and holistic. The proposed study will serve as an opportunity to empirically refine our theoretical framework.

### Study Aims

The aims of this study are to (1) decode and understand the ways in which BSMCs express their romantic relationships in virtual contexts via symbolic indicators, (2) determine how the couples describe the quality of their romantic relationships, and (3) explore how the couples make meaning of their relationship quality and its impact on their relational and individual health.

## Methods

### Ethical Approval

This study received ethical approval and oversight from the Rowan University Institutional Review Board on October 8, 2020 (IRB-FY2021-7).

### Setting and Participants

All study procedures will be remote and take place on online platforms (ie, Facebook, Instagram, Twitter, and Zoom videoconferencing). Only BSMCs will be recruited for this study. Both partners in the couple must self-report (1) being at least 18 years old; (2) having been assigned male sex at birth; (3) identifying as a Black (meaning of African descent) American man; (4) currently being in a committed (at least 3 months long) romantic relationship with each other [9], in which “committed” is taken to mean both partners consider their partner above anyone else and the relationship has been sexual [29]; (5) having access to a reliable high-speed internet service that allows for videoconferencing; (6) being able to proficiently speak and comprehend English; (7) being willing to participate in both the initial interview and the follow-up member checking interview; and (8) being able to provide informed consent. Each partner must also provide (via picture or video) a negative test result for HIV from within the last 6 months. Both partners are required to agree to participate in both interviews together at the same time.

Couples will be excluded if (1) at least one partner presents with severe intoxication at the time of screening or the interview; (2) at least one partner presents with active psychosis at the time of screening or the interview; (3) at least one partner reports current, ongoing intimate partner violence within the relationship; or (4) the couple breaks up between the first and second interviews. Couples who meet all inclusion criteria and have been determined eligible but meet exclusion criteria 1 or 2 will be offered the chance to complete their interviews at a later time (no more than 2 weeks later), when intoxication or mental health symptoms do not interfere with their cognitive and behavioral abilities. Couples who meet exclusion criteria 3 or 4 will be deemed ineligible for further participation in the study.

### Study Design

**Figure 2:**
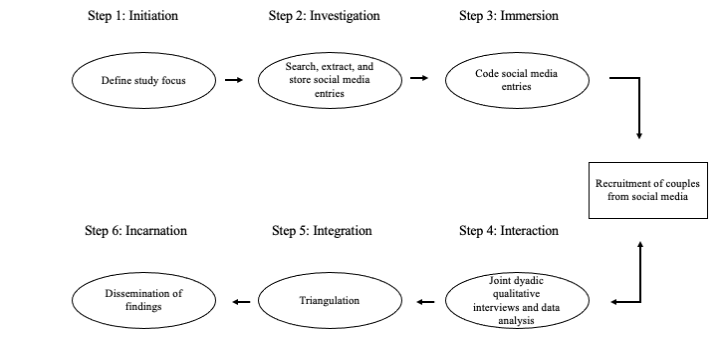
Overview of netnography study procedures.

This study utilizes two different qualitative methods, symbolic netnography and joint dyadic interviewing, to achieve its aims. The findings from symbolic netnography inform the data collection procedures for the joint dyadic interviews, which are embedded within the netnography design. Findings from both qualitative data collection methods will be integrated using Farmer and colleagues’ triangulation protocol [30] to explore convergence and divergence between them. An overview of the study procedures is depicted in Figure 2.

Symbolic netnography is a form of qualitative research that analyzes a specific group’s cultural experiences as reflected in their online (eg, social media) presentations and interactions [18]. Such analyses are used as building blocks to understand people’s behaviors, meaning-making strategies, values, and decision-making processes. This form of inquiry offers an opportunity to understand participants in contexts they control and in their own terms across presentation modalities (eg, pictures, text, sound, and video). This is especially important for BSMCs given the decreasing availability of physical social spaces (eg, bars and clubs) exclusively for Black sexual-minority men. Gentrification has contributed to venue owners who are Black sexual-minority men not being able to afford their rents [31]. Additionally, the unforeseen social realities of a post–COVID-19 world have complicated Black sexual-minority men’s ability to gather in culturally affirming physical spaces [32].

Joint dyadic qualitative methodology assumes that a dyad’s reality is coconstructed and thus utilizes qualitative interviewing with both members of a dyad [33]. In our study, couples will be interviewed together at the same time so that we can gain an understanding of how they create a joint picture and shared narrative of their relationship and its meaning. The joint dyadic qualitative interviewing is nested within the symbolic netnography. Overall, this methodology will ensure that the knowledge we discover is couples-based and directly applicable to couples-level health.

### Symbolic Netnography

The symbolic netnography follows the six steps outlined by Kozinets [18]. These guidelines include: initiation, investigation, immersion, interaction, integration, and incarnation.

#### Initiation

This step encompasses the specification of the study’s focus. The focus of this study is BSMCs’ online presentations of their romantic relationships and the symbolic indicators that provide information about the quality of those relationships. Specifically, we are interested in the following symbolic indicators: terms of endearment, relationship commitment, physical affection, social performance of the romantic relationship, expressions of care, and love languages [34] (eg, verbal and nonverbal communication of care for one’s romantic partner via such acts as providing words of affirmation, gifts, and physical touch). We will also examine how social support for the couple is expressed on social media.

#### Investigation

At the investigation step, we will search social media databases and select BSMCs’ entries that present images, text, audio, and video displaying symbolic indicators of their romantic relationships. We have chosen Facebook, Instagram, and Twitter as the social media sites that comprise the landscape of this study. These sites were chosen due to high rates of use among Black sexual-minority men [35,36]. We will use an initial list of 29 hashtags to conduct our search for BSMCs’ entries. These hashtags were developed through individual interviews with a convenience sample of 20 Black sexual-minority men who were active on social media. Examples of hashtag search terms include #blackgayweddings, #blackm4m, and #blackmenlovingblackmen. We will also utilize new hashtags that we discover in the process of our initial search to conduct subsequent searches. We expect to find a large number of entries and will use theoretical sampling (ie, sampling for entries that reflect concepts related to our integrated Afrocentric interdependence theoretical framework, such as intimacy, commitment, spiritual consciousness, and Black cultural pride) to select which entries will be included in the final dataset. We will read, watch, and listen to the information presented in the entries. Social media entries will be included if they (1) are composed of images, text, audio, or video; (2) are in the English language; (3) depict a BSMC; and (4) are created by the couple and not a profile or page that highlights other couples only. Social media entries will be excluded if they are (1) videos uploaded by government, professional, educational, or news organizations; (2) entries about someone else’s romantic relationship; or (3) entries with product or commercial endorsements.

We will continue to archive entries until saturation (ie, no new information is discovered) is reached for each concept. Entries will be saved via screen captures. We will redact screen names from all entries and store them in a separate, secure dataset (described below). All entries will be given an ID number that corresponds to the screen name in the separate database. The deidentified entries will be stored in an NVivo database in a password-protected folder on the principal investigator’s secure drive.

#### Immersion

In this stage, we will review the social media entries in the NVivo database and record our reflections (via memos) about our impressions of each entry. For example, for each entry we will reflect on (1) emotions that surfaced when coding the entry, (2) who this couple is, and (3) personal biases and assumptions about the entry. This reflection process will help us cultivate “high quality...‘deep’ data” [18] that illustrates the couples’ cultural experiences of their romantic relationships. These reflections will be compiled in an immersion journal. Next, we will analyze the social media entries via coding. We have created a coding guideline protocol that we will use to assess the symbolic indicators of relationship qualities (ie, terms of endearment, relationship commitment, physical affection, social performance of the romantic relationship, expressions of care, and love languages). This coding protocol was informed by our integrated Afrocentric interdependence theoretical framework. We will use the coded social media entries to inform refinement of our integrated Afrocentric interdependence theoretical framework to better explain BSMCs’ romantic relationship quality and expressions.

We will also create a separate dataset with the social media entries’ screen names and the emergent theoretical concepts associated with each entry. The screen name dataset will serve as a directory (ie, participant pool). The concepts in the emergent theory will guide theoretical sampling from the screen name dataset. We will invite couples from the screen name dataset to participate in joint dyadic qualitative interviews until we reach saturation on each theoretical concept. In this way, the process is iterative. Based on previous research, we expect to reach theoretical saturation by 20 interviews. However, if new concepts continue to emerge, we will continue to invite couples for interviews and analyze their data until saturation is reached.

#### Interaction

Research assistants will send private messages to contacts via the social media site. The research team will create 3 social media accounts, 1 for each social media platform: Twitter, Instagram, and Facebook. All accounts will be linked to the study email address that we will register with Gmail. All usernames will be transparent and include the following term: “#blackgaylove Research Team.” When contacting potential participants from the screen name dataset, we will immediately identify ourselves as researchers who are recruiting for a research study. If a potential participant declines, we will not contact them again. If a potential participant does not respond immediately, we will reinitiate contact in 7 days. If a potential participant does not respond after the second contact attempt, we will not contact them again. We do not anticipate any major disruptions into the social media culture, as messages will be private and thus can be easily ignored. We will not contact a potential participant more than twice in the recruitment process to avoid coercion and minimize frustration.

Initial contact on social media will be guided by the following script: “Hello, my name is [research team member’s name]. I am a part of the #blackgaylove Research Team. We’re a team headed by two Black gay men and we’re interviewing Black gay male couples about their relationship qualities. Would you be interested in learning more about this study? If eligible, you and your partner can earn $60 each for your time.”

The partner who responds initially (the index partner [IP]) will be asked for his email address and phone number. Then he will be asked to share the other partner’s (the nominated partner [NP]) contact information. A member of the research team will then contact the IP and NP via email and invite them to be screened for the study. After both the IP and NP of each couple have communicated interest in being screened for the study, a research team member will contact each partner separately to assess eligibility and obtain informed consent. Each partner will be asked to provide consent separately to encourage him to ask questions about the research process candidly and fully and to ensure that one partner does not coerce the other into enrolling.

After each partner has been screened, deemed eligible, and provided informed consent, this will be documented in the contact database, where all participant contact information is stored. At this time, each partner will be given an ID number that will be used to identify his data. This will be used in lieu of names as a measure to ensure confidentiality. Partners will then be emailed jointly (ie, the email will be addressed to both partners) with a Calendly link where they will sign up for a two-hour time slot. They will also be emailed a link to a short demographic questionnaire, accessed via Qualtrics, that they will complete before their interview. At the time of the interview, a member of the research team will assess the identity of each partner and ensure that both are present. At this time, they will also be asked to provide visual proof of a negative test result for HIV (via picture or video) from within the last 6 months. After this evaluation, the interview will be conducted and recorded via a Health Insurance Portability and Accountability Act (HIPAA)-compliant version of Zoom. All recordings will be stored locally on the research team members’ computers and not stored in the Zoom cloud. Consistent with our prior work, each partner in the couple will receive $30 for their completion of each interview for a total of $60 each.

Participants in this study will be expected to complete 2 dyadic qualitative interviews. The first interview will last for no longer than 2 hours. The second interview, which will take place on a separate day, is expected to last no longer than an hour and a half. The second interview will be conducted after each couple’s first interview has been transcribed and analyzed.

#### Joint Dyadic Qualitative Interviews

We will use the critical incident technique (CIT), which is an investigative tool to uncover existing realities or truths so they can be measured and predicted. CIT prompts participants to recall a specific, concrete incident in which the phenomenon under study was activated in their consciousness and influenced their experience of the world around them [37]. The technique gives participants freedom in describing the experience and assumes that participants will share incidents that have high priority and that have been most impactful for them. Couples will be asked to tell a series of stories about incidents when they demonstrated (1) love for each other; (2) they cared for each other; (3) how committed they were to each other; and (4) a public display of their romantic relationship. They will also be asked about social support for their romantic relationship that they have received from others, as well as how their romantic relationship has influenced their self-conception, health, and their lives overall.

#### Integration

After joint dyadic qualitative interviews are completed and analyzed, we will integrate the data from the 2 qualitative methods using Farmer et al’s [30] triangulation protocol, which includes creating a convergence/divergence matrix to assess similarities and differences among datasets (ie, social media data and interview transcripts) and findings. We will assess coverage of codes across datasets. Then, we will conduct peer debriefing and member checking to ensure credibility and trustworthiness of the data. Member checking will be conducted by inviting participants who completed the dyadic qualitative interviews to participate in a brief follow-up interview. Specifically, we will call them and ask them to reaffirm consent to a second interview. After obtaining and documenting verbal consent, we will email each member of the couple a list of the research team’s findings and ask them to review them. No more than 7 days later, we will call them via Zoom and ask them (1) whether the findings make sense to them, (2) how well their experiences are adequately represented by the findings, and (3) whether they believe some findings should be modified and how should they be modified.

Peer debriefing will include eliciting feedback from 5 academic colleagues (to be determined) at Rowan University. These colleagues will be experienced qualitative researchers who are not in the Psychology Department at Rowan University (the first author’s home department) and who are unfamiliar with this study. The colleagues will be emailed the findings and transcripts and asked to assess them for over- and under-emphasized points, errors, and bias. These colleagues will be asked to respond via email to the first author with written feedback within 14 days of receipt of the findings and transcripts. All feedback will be reviewed by the research team and used to inform subsequent interpretations of the data.

#### Incarnation

This step involves disseminating the netnography findings in traditional outlets (eg, peer-reviewed journals and conference abstracts) and nontraditional outlets (eg, social media, organizational websites, and listservs). For example, in addition to publishing peer-reviewed articles, we will also create infographics describing our findings. We will share them on Instagram, Twitter, and Facebook. Social media entry data will not be shared (unless participants give explicit permission) to ensure participant privacy. Joint dyadic interview data will be reported as quotes attributed to participants’ pseudonyms to protect confidentiality.

### Data Analysis

Guided by Corbin and Strauss’s recommendations [17], data will be analyzed using a grounded theory approach, which provides systematic methods and strategies for inductively analyzing data aimed toward theory development, and in this study, toward theory refinement. We will review a small set of transcripts (n=6) to develop a codebook. By employing grounded theory methods, transcripts will be parsed into meaningful units of information using line-by-line analysis, constant comparative analysis, and open coding [17]. We will use memos to describe code properties and dimensions and organize them into a codebook. The codebook will include the name of each code, the definition of each code, and an excerpted quote that illustrates each code. Research assistants will use the codebook to code the remaining transcripts and utilize memos to document emergent codes and discrepancies in coding. Memos will be used to keep track of analysis decisions and insights as well as to record the coder’s emotions, impressions, and responses during the research process. After the initial coding of all transcripts is complete, κ statistics [38] will be used to compare results between coders. Inconsistent codes and any other discrepancies will be discussed and revisions will be made until coders obtain 90% agreement. Data collection and analysis will occur simultaneously, as is customary with grounded theory methodology. After all coding of interviews is complete, we will then code memos using the process described above.

We will refine our integrated Afrocentric interdependence theoretical framework using our codes as the building blocks. Specifically, we will compare codes across participants for similarities and differences. We will integrate similar codes into larger categories. Then, we will organize these larger categories into a core category—“a concept that is sufficiently broad and abstract that summarizes in a few words the main ideas expressed in the study” [17]. Next, we will integrate all the attributes and dimensions of the other categories into the core category. Finally, we will use memoing to develop the main descriptive story of our refined theory and construct an integrative diagram to depict it visually.

Our use of a grounded theory approach in conjunction with a directive theoretical framework and codebook may seem counterintuitive to some readers. However, these strategies are not mutually exclusive and align well with Corbin and Strauss’ [17] recommendations for theory development and refinement. The authors wrote: “Though a theory that comes from data is grounded, the final ‘theory,’ or how concepts fit together, is constructed by the researcher...depending upon the perspective of the researcher and where he or she decides to put the emphasis” [17]. Thus, our use of a grounded theory approach allows us to refine our integrated Afrocentric interdependence theoretical framework—which informs our conceptual perspective—with empirical data that emerges from analysis of the netnography and joint dyadic interview data.

## Results

Data collection began for this study in November 2020 and results are expected to be available no later than December 31, 2022.

## Discussion

### Principal Findings

This is a protocol for a study that seeks to (1) decode and understand the ways in which BSMCs express their romantic relationships in virtual contexts via symbolic indicators; (2) determine how the couples describe the quality of their romantic relationships; and (3) explore how the couples make meaning of their relationship quality and its impact on their relational and individual health. We expect 3 research products to be developed from this study. The first is a netnography dataset that will be analyzed to understand the couples’ expression of their romantic relationships online. The second is a secure database of social media screen names of BSMCs that will facilitate recruitment of such couples in this study and possibly future studies. The third is a refined integrated Afrocentric interdependence theory that describes and explains BSMCs’ romantic relationship quality and health. This theory will be used by the research team to design future couples-based health research that focuses on the distinct lived experiences and health concerns of the couples. The theory may also provide an innovative and culturally specific framework to guide other researchers who are interested in working with BSMCs in a culturally informed manner.

### Potential Limitations and Alternatives

This study poses no physical risk and only minimal risk of psychological stress or harm. To protect participants, they will be provided a list of names and telephone numbers of several nationally available mental health hotlines and mental health organizations to discuss any distress they may experience. The referrals are free of charge and any follow-up fees for services beyond the first consultation will be the responsibility of the participant. Participants will be told that they can decline to answer any questions and encouraged to discontinue their participation at any time if they feel inordinate psychological or physical distress. Participants will also be informed that they have the right to withdraw their data at any time, by requesting, in writing, that it be removed from the dataset. Retaining participants between interviews is also a concern that we will address by collecting detailed contact information and providing reminders (ie, calls, emails, and text messages) to increase attendance for both interviews [39].

If a couple breaks up between the initial joint dyadic and member checking interviews, each partner will be invited to complete a break-up assessment—this has been used in our prior work [39]. The break-up assessment will query and document when the couple broke up and whether participation in the study contributed to the break-up. Then, we will provide free-of-charge referrals to psychological services (eg, hotlines and contact information for low-cost therapists). After completing the break-up assessment, the couple will be unenrolled from the study and study staff will no longer contact them. In our prior work, there was 20% attrition due to break-ups over a 9-month period. Given that our study period is much shorter (ie, 1 month), we anticipate a significantly lower break-up rate.

Finally, we recognize that our sample will be restricted to those with an active relevant social media presence. Our protocol does not reach couples who are not active on social media or whose social media presence does not present a public display of their romantic relationships (but who nonetheless would have perspectives to offer). However, our study serves as an important starting point for assessing romantic relationship quality among BSMCs, in a space that they control, to inform future couples-based health interventions and policy.

### Conclusion

This study will apply novel symbolic netnographic qualitative methods to add to an understanding of BSMCs’ romantic relationships and how they contribute to their health. Through a symbolic netnographic exploration of the couples’ romantic relationships, this study will access a largely underexplored source of intersecting racial and sexual minority cultural data and symbolic relationship indicators that will be collected and analyzed in a naturalistic and holistic manner—in the social context of the couples’ online presence. Further, contextualizing the couples’ online data and relationship indicators in this way treats the social act of being in a BSMC as a crucial unit of analysis that can render a rich portrayal of their online social presence and interactions as more explanatory (rather than being merely descriptive) of how their relationship quality plays a part in their health as individuals and as couples. Moreover, the findings from this research project will be used to develop programs to improve BSMCs’ health in community and virtual settings. It may also be used to influence policy that advocates for Black sexual-minority men’s health.

## Abbreviations

BSMC: Black same-sex male couple
CIT: critical incident technique
IP: index partner
NP: nominated partner
OCT-SGM: Optimal Conceptual Theory applied to sexual and gender minorities
SGM: sexual and gender minority
